# Supplementary material for: Layer-specific integration of locomotion and sensory information in mouse barrel cortex
Source: Nat Commun. 2019 Jun 13;10:2585. doi: 10.1038/s41467-019-10564-8 (PMC6565743; doi:10.1038/s41467-019-10564-8)
Supplement: Supplementary file 1 — Supplementary information [file 41467_2019_10564_MOESM1_ESM.pdf]

Supplementary Information

Supplementary Figure 1

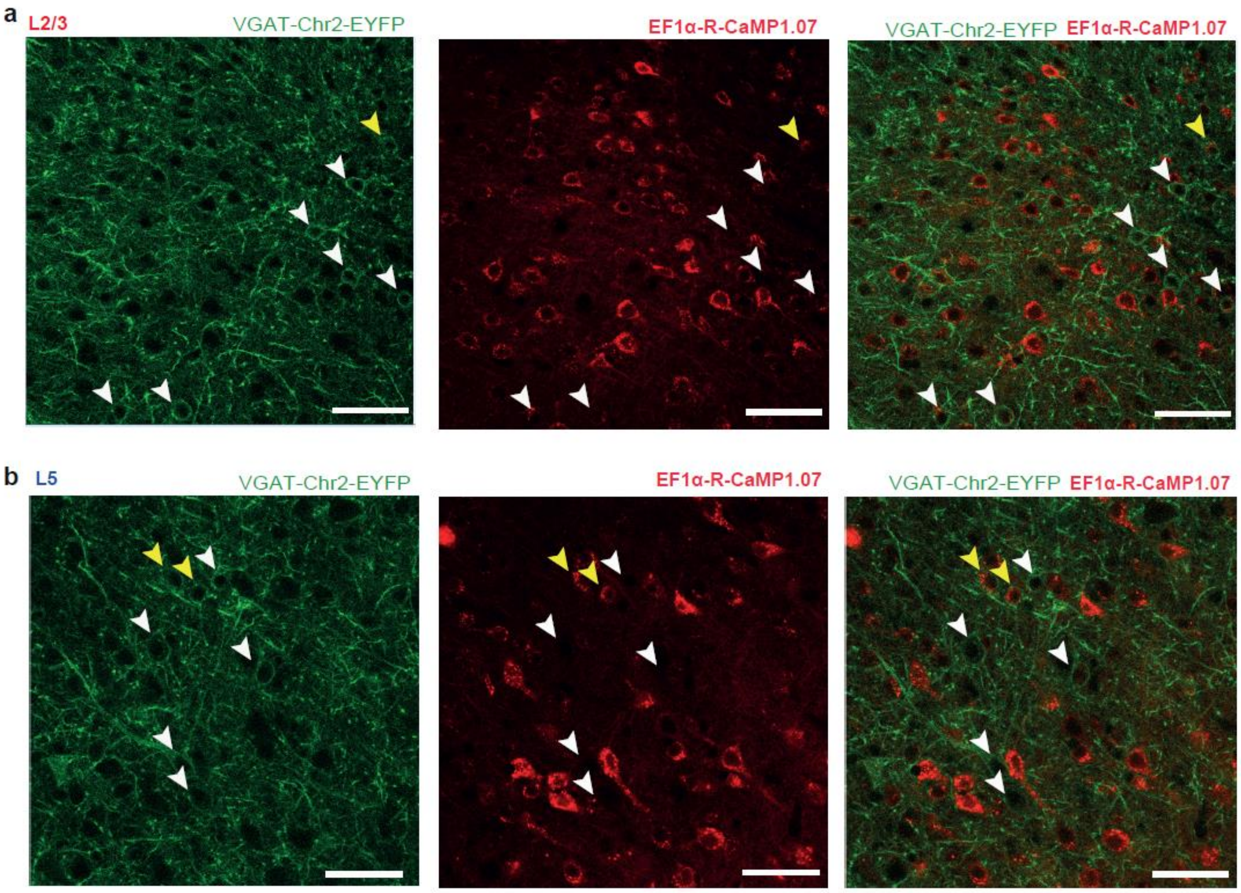

Supplementary Figure 1. Viral expression of R-CaMP1.07 mainly labels pyramidal neurons in mouse barrel cortex.

(**a**) We injected AAV2.1-EFα1-R-CaMP1.07 into barrel cortex of transgenic mice expressing ChR2-EYFP in GABAergic neurons. Left panel shows expression of EYFP in cell membrane and projections of GABAergic neurons in L2/3 of a coronal slice from barrel cortex. Middle, is the expression of R-CaMP1.07 in the same section. On the right is the merged view. All arrowheads point to EYFP expression of somatic membrane of GABAergic neurons, yellow arrowheads show co-expression of R-CaMP1.07. (**b)** Expression of EYFP (left, green), R-CaMP1.07 (middle, red) and merged view (right) from a cross section from L5 of the barrel cortex. In total we counted 570 R-CaMP1.07 expressing neurons in L2/3 of 3 coronal slices from 2 mice, of which 19 (3.3%) were also EYFP positive. In two cross sections of L5, there were in total 178 R-CaMP1.07-positive neurons, of which 11 (6.2%) were EYFP positive. Of all EYFP-positive neurons 35% and 39% showed R-CaMP1.07 expression in L2/3 and L5, respectively. All scale bars are 50 µm.

Supplementary Figure 2

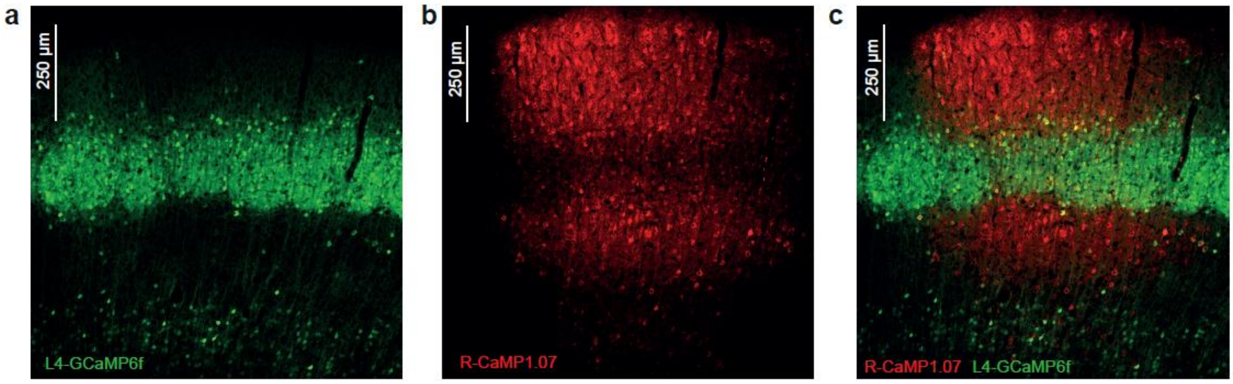

Supplementary Figure 2. Viral expression of R-CaMP1.07 mainly targets L2/3 and L5 neurons in mouse neocortex.

We injected AAV2.1-EFα1-R-CaMP1.07 throughout the barrel cortex (at 800-200 µm depth) of triple-transgenic mice expressing GCaMP6f in L4 (Nr5a1-Cre;ROSA26-ZtTA;Ai93(TITL-GCaMP6f)). (**a**) Expression of GCaMP6f in L4 neurons in a coronal slice from barrel cortex. Some scattered expression is visible in deeper layers. (**b)** R-CaMP1.07 expression in the same coronal slice, showing strong expression in superficial and deep layers and the typical expression gap in L4. (**c**) Overlay of (a) and (b) Co-expression of R-CaMP1.07 and GCaMP6f occurs very rarely in only few cells. R-CaMP1.07-expressing L5 neurons are clearly recognizable below L4.

Supplementary Figure 3

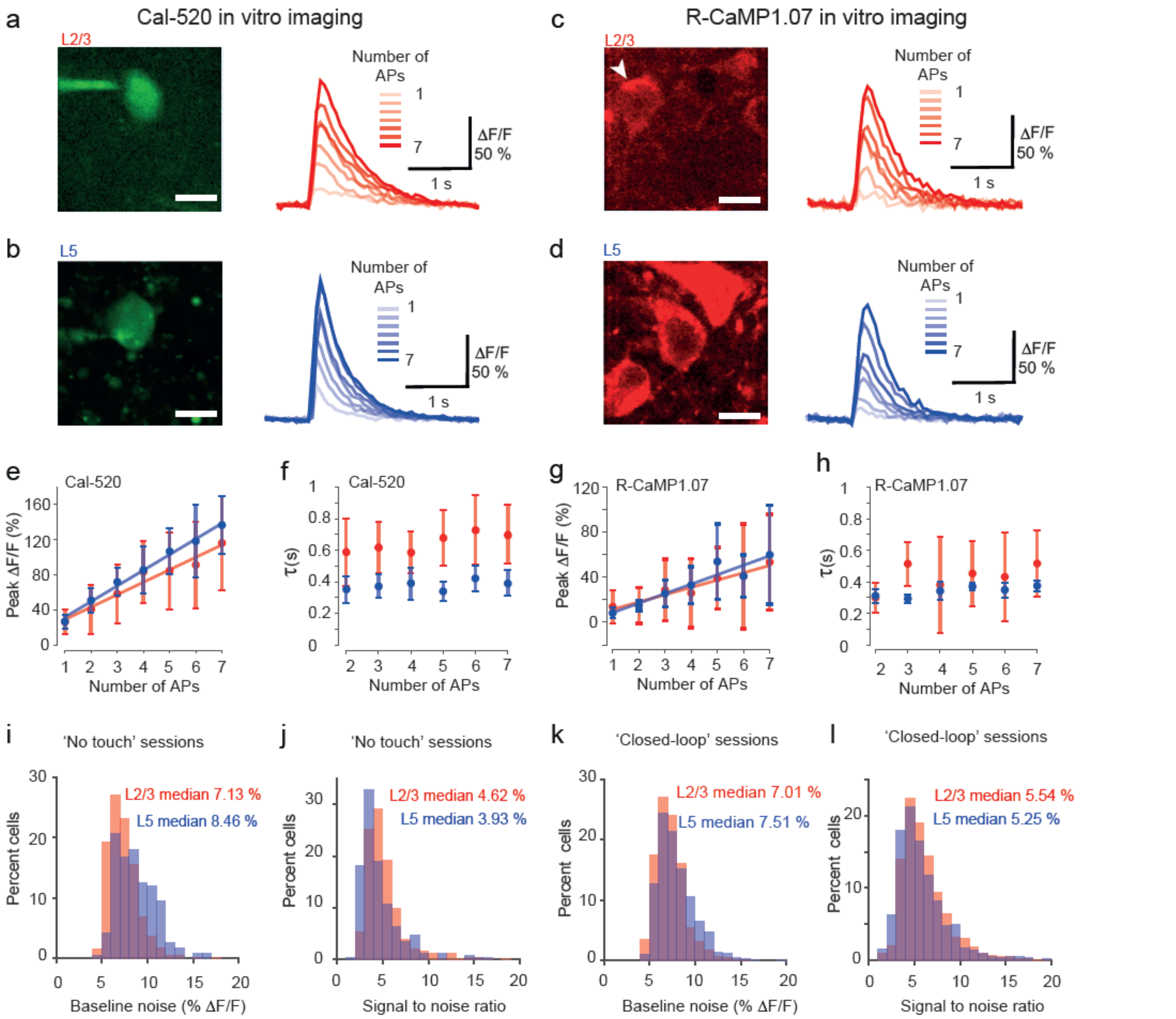

Supplementary Figure 3. Comparison of action potential-evoked calcium transients in L2/3 and L5 pyramidal neurons.

(a) Left: Two-photon image of example L2/3 neuron filled with Cal-520 through a patch pipette in vitro. Right: mean ΔF/F calcium transients evoked by 1-7 action potentials induced by current injections. Scale bars indicate 10 µm in panels **a-d**. (**b**) Same as **a** but for an example L5 neuron. (**c**) Left: Two-photon image of example L2/3 neuron expressing R-CaMP1.07 in vitro. Right: mean ΔF/F calcium transients evoked by 1-7 action potentials induced by current injections. (**d**) Same as **c** but for an example L5 neuron. (**e**) Mean peak ΔF/F amplitudes (± s.d.) as a function of the number of recorded action potentials in Cal-520-filled neurons (n = 4 L2/3, red; n = 5 L5, blue). (**f**) Decay time constant (τ) of Cal-520 ΔF/F calcium transients as a function of number of action potentials (overall mean 650 ms for L2/3 neurons, red, and 378 ms for L5 neurons, blue; p=0.016, Wilcoxon rank sum test). (**g**) and (**h**) Same as **e** and **f** but for 4 L2/3 neurons and 3 L5 neurons expressing R-CaMP1.07 in 4 mice (mean τ 438 ms and 340 ms for L2/3 and L5 neurons, respectively; p = 0.4, Wilcoxon rank sum test). (**i**)-(**l**) Comparison of in vivo R-CaMP1.07 signals for two experimental conditions. (**i**) Distribution of baseline noise (Methods) for all neurons in all 'No-touch' sessions (705 L2/3 neurons, red, and 233 L5 neurons, blue). (**j**) Distribution of signal-to-noise ratio (Methods) for all neurons during No-touch sessions (L2/3, red; L5, blue). (**k**) and (**l**): same as **i** and **j** but for all neurons imaged in 'Closed-loop' sessions (1088 L2/3 neurons, red, and 728 L5 neurons, blue).

Supplementary Figure4

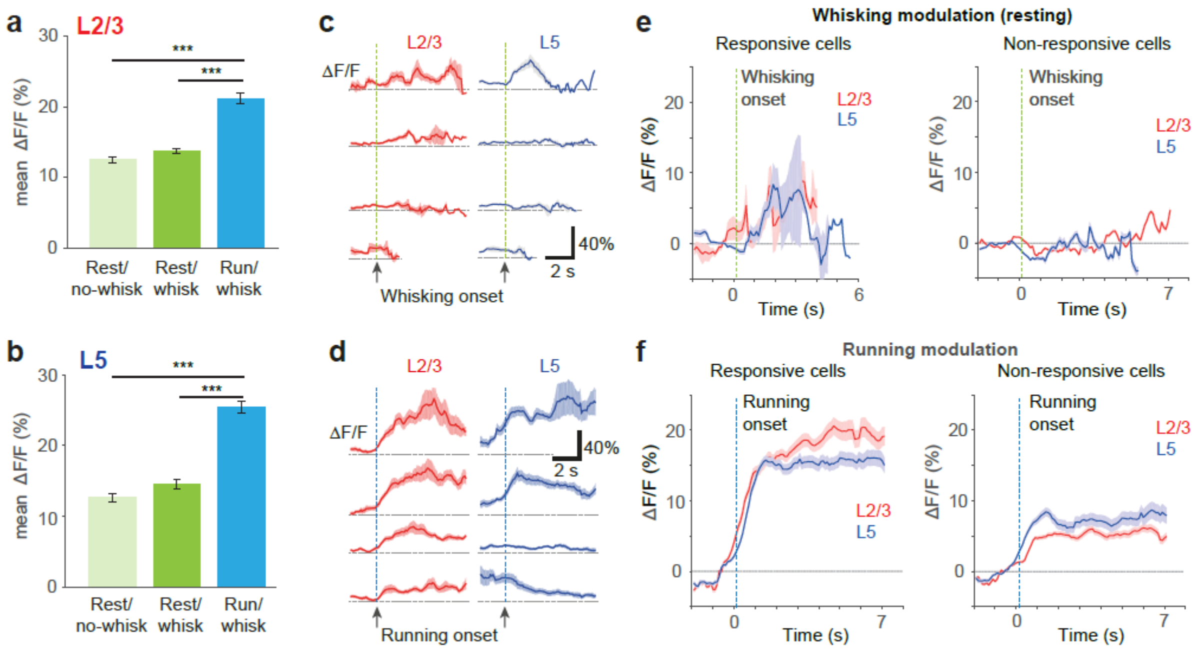

Supplementary Figure 4. Running increases neuronal activity in both L2/3 and L5 of S1.

(**a**) Comparison of mean ΔF/F values (± s.e.m.) for L2/3 neurons during periods of resting/no-whisking, resting/whisking, and running/whisking in the absence of any texture stimulus (12.5 ± 0.4%, 13.7 ± 0.4% and 20.8 ± 0.7%, respectively; n = 342 neurons). (**b**) Same as **a** for L5 neurons. Mean ΔF/F values: 12.7 ± 0.6% , 14.5 ± 0.7%, and 25.4 ± 0.9% for the three behavioral states (n = 168 neurons). Statistical significance in was computed with multi-sample comparison with one-way ANOVA (*** $p < 10^{-9}$; p = 0.21 [L2/3] and 0.19 [L5] for resting/no-whisking versus resting/whisking). (**c**) Average ΔF/F traces of example neurons aligned to whisking onset (green vertical dotted line) during resting periods (shading shows ± s.e.m.). Only the period of continued whisking after onset was considered for each trial, hence the variable lengths of traces for different neurons. (**d**) Similar to **c**, calcium signal of example neurons aligned to running onset (blue vertical dotted line). Only the period of continued running after onset was considered for each trial (shading indicates ± s.e.m.). (**e**) Comparison of mean ΔF/F transients aligned to whisking onset for responsive (left) and non-responsive (right) neurons. 11/342 L2/3 neurons and 14/168 L5 neurons showed significant responses to whisking onset. Responsive neurons displayed ΔF/F amplitudes larger than two times the baseline noise σ (Methods). (**f**) Similar to **e** but comparing mean ΔF/F transients aligned to running onset for responsive (left) and non-responsive (right) neurons. 108/342 L2/3 neurons and 64/168 L5 neurons showed significant responses to running onset. The increase in activity in non-responsive neurons indicates that even larger populations show up-modulation, which however did not reach significance due to our relatively conservative selection criterion (> 2σ).

Supplementary Figure 5

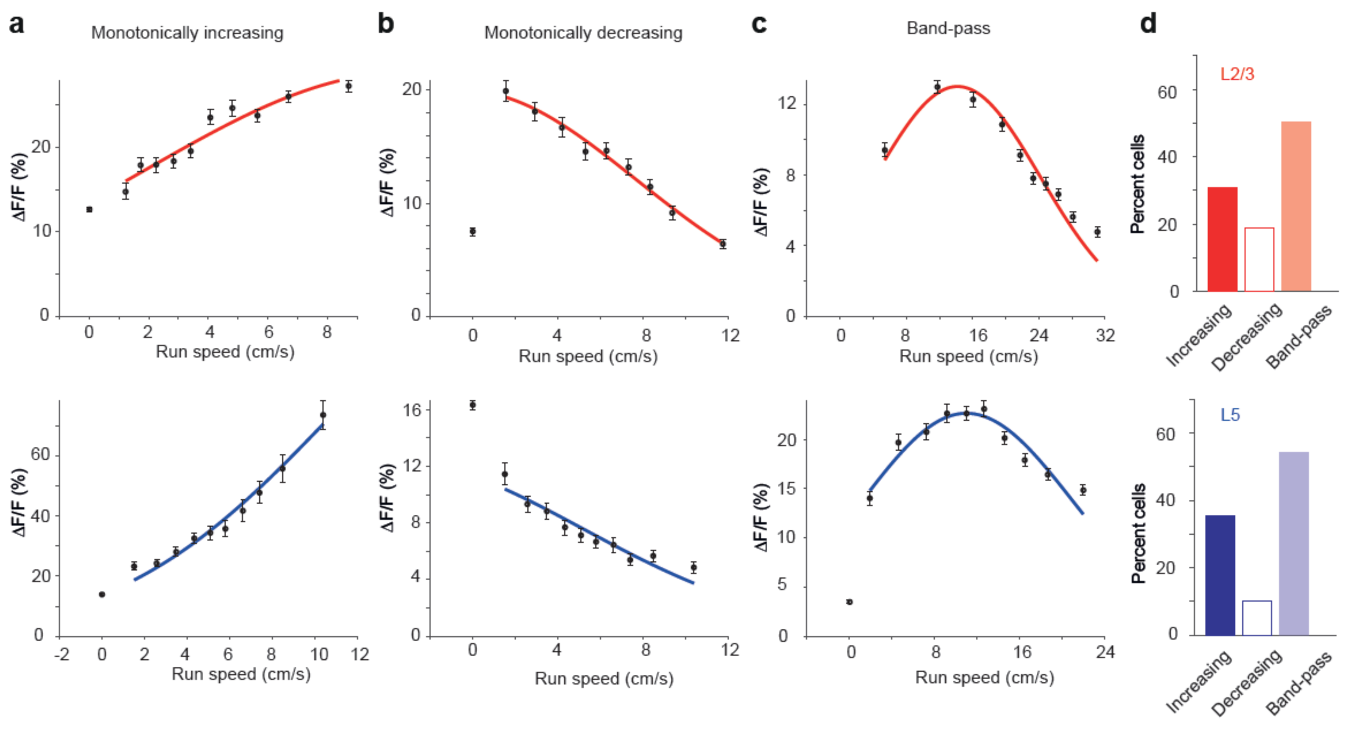

Supplementary Figure 5. Run-speed tuning of S1 neurons in the absence of wall-touch.

(**a-c**) Dependence of ΔF/F signal on run speed for 6 example S1 neurons measured during 'No-touch' sessions. (**a**) Example L2/3 neuron (top) and example L5 neuron (bottom) with monotonically increasing run-speed tuning. (**b**) Example L2/3 neuron (top) and example L5 neuron (bottom) with monotonically decreasing run-speed tuning. (**c**) Example L2/3 neuron (top) and example L5 neuron (bottom) with band-pass run-speed tuning. (**d**) Percent proportion of L2/3 neurons (top) and L5 neurons (bottom) neurons with monotonically increasing, monotonically decreasing and band-pass run-speed tuning. Only neurons with significant run-speed modulation were considered (276/705 L2/3 neurons and 107/233 L5 neurons; Methods).

Supplementary Figure 6

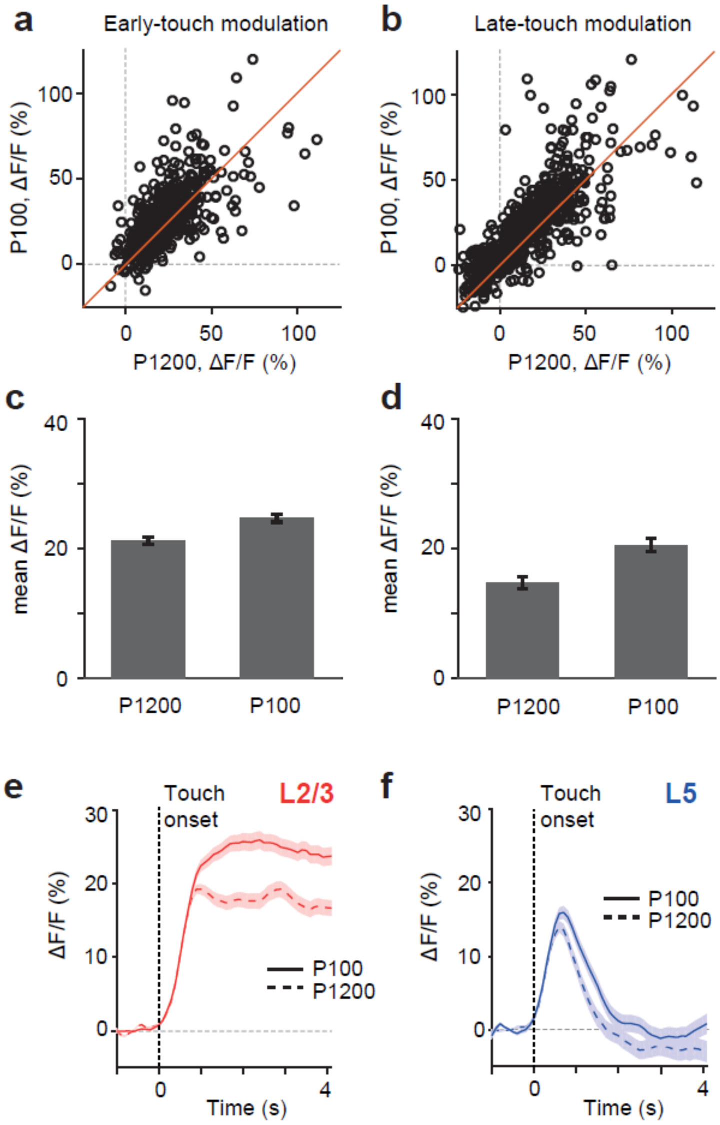

Supplementary Figure 6. Wall-touch responses to different textures.

We used sandpapers of two different graininess (P100-rough and P1200-smooth) in our touch experiments. Sessions either involved a single texture or random representations of both textures in each trial. Here we compare neuronal responses to wall touch of random presentation of two different textures (14 Closed-loop sessions, n = 691, neurons might be repeated). (**a**) Scatter plot of early-touch modulations of wall touch events where the wall was textured with P1200 (x-axis) and P100 (y-axis) sandpaper. (**b**) Same as in **a** but comparing late-touch modulations. (**c**) Population averages of early-touch modulations elicited by smooth and rough textures, respectively (21.4 ± 0.5% ΔF/F vs. 24.9 ± 0.6% ΔF/F, mean ± s.e.m., $p < 10^{-4}$ paired T-test). (**d**) Same as in **c** but for late-touch modulations (15.0 ± 0.9% ΔF/F for P1200 vs. 20.9 ± 1.0% ΔF/F for P100, mean ± s.e.m. , $p < 10^{-4}$ paired T-test). (**e**) and (**f**) Comparison of population averages of touch responses for rough (P100) and smooth texture (P1200) for L2/3 (n = 465) and L5 (n = 226) neurons, respectively. Although the rough texture elicited a slightly larger responses, consistent with previous findings[1], the difference in sustained versus transient touch responses of L2/3 and L5 neurons was not affected by texture identity.

Supplementary Figure 7

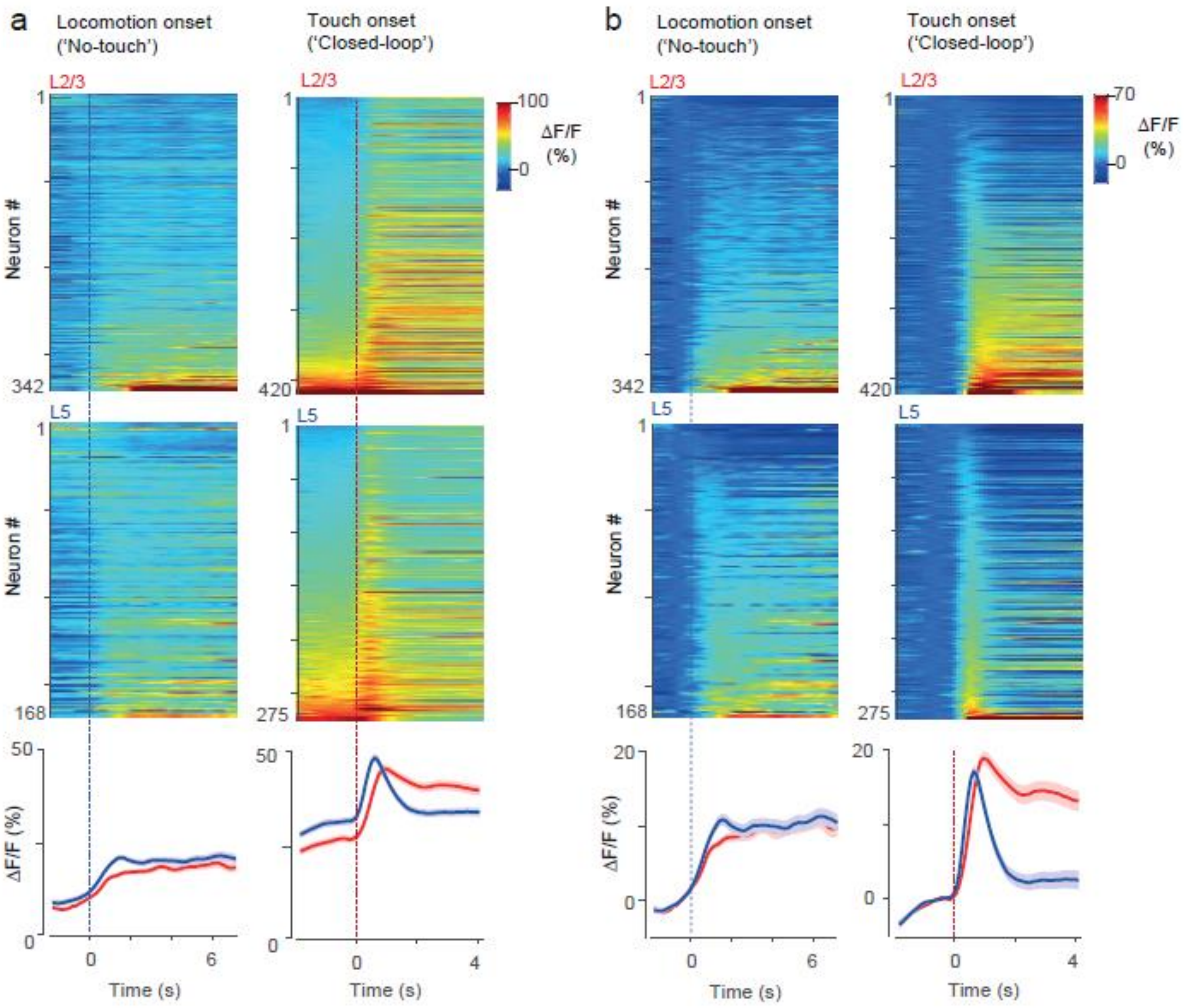

Supplementary Figure 7. Population response of locomotion onset and touch onset responses in S1. (**a**) Left: The heat maps represent locomotion onset responses of all L2/3 (top) and L5 neurons (middle) in No-touch condition. Each row represents the fluorescence change of a single neuron. Locomotion onset time point is shown in blue dotted line. Population average of locomotion onset responses is shown in the bottom panel (L2/3 red, L5 in blue). Pre-onset activity is not subtracted in these plots. Right panels: The heat maps represent touch onset responses of all L2/3 (top) and L5 neurons (middle) in Closed-loop condition. Bottom panel shows population averages. Red dotted line indicates texture moving in touch with whiskers. Neurons are sorted according to their pre touch-onset activities. High pre-touch activity indicates increased response due to locomotion as Closed-loop experiments required animals to move a certain distance before texture touch. (b) Locomotion and touch-onset responses of all L2/3 and L5 neurons similar to panels shown in (**a**). Responses in **b** are computed by subtracting the mean activity during a pre-onset window (-1 to -0.3 s).

Supplementary Figure 8

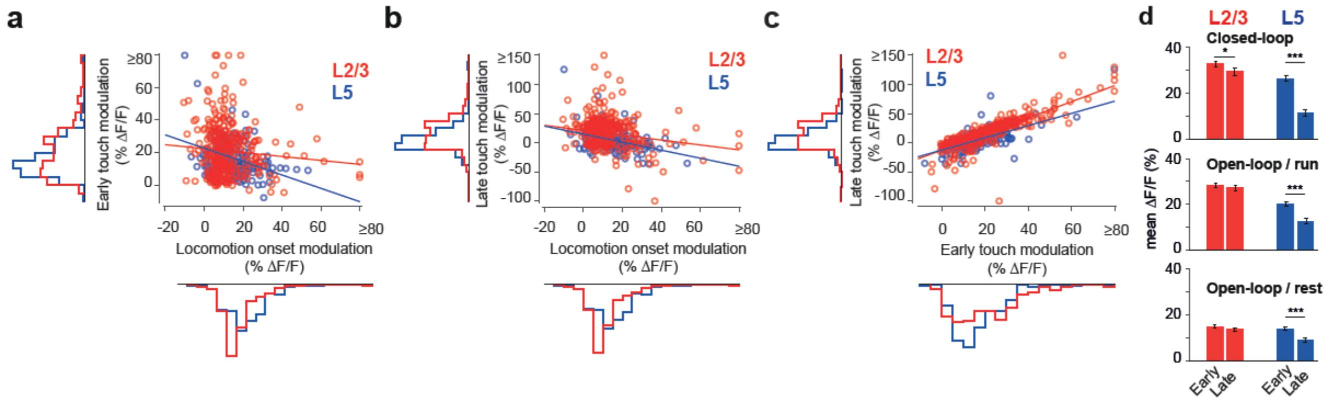

Supplementary Figure 8. Comparison of relations between locomotion onset, early touch and late touch modulations.

(**a**) Locomotion onset modulation vs early touch modulations (**b**) Locomotion onset modulation vs late touch modulations (**c**) early touch vs late touch modulations. L2/3 neurons are in red and L5 neurons are in blue. Only neurons which were imaged both in ‘No-touch’ and ‘Closed-loop’ sessions were considered. Neurons may be represented more than once if they were imaged in multiple sessions. (**d**) Comparison of early and late phase touch responses for L2/3 (red) and L5 (blue) populations under 3 conditions: Closed-loop (top panel), Open-loop during running (middle panel) and Open-loop during resting (bottom panel; paired T-test, * $p = 0.003$, *** $p < 10^{-4}$).

Supplementary Figure 9

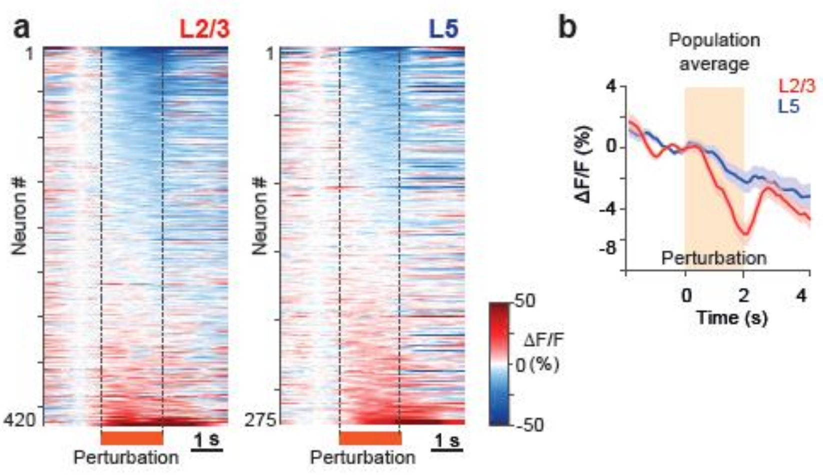

Supplementary Figure 9. Decrease in activity dominates population responses to mismatch of running speed and tactile flow.

(**a**) The heat map represents perturbation-evoked responses of all L2/3 (left column) and L5 neurons (right column) in Closed-loop condition. Each row represents the fluorescence change of a single neuron compared to the mean activity during a pre-perturbation window (-1 to -0.3 s). Orange period is when tactile flow was stalled suddenly. (**b**) Population average of all neurons aligned at perturbation onset.

Supplementary Figure 10

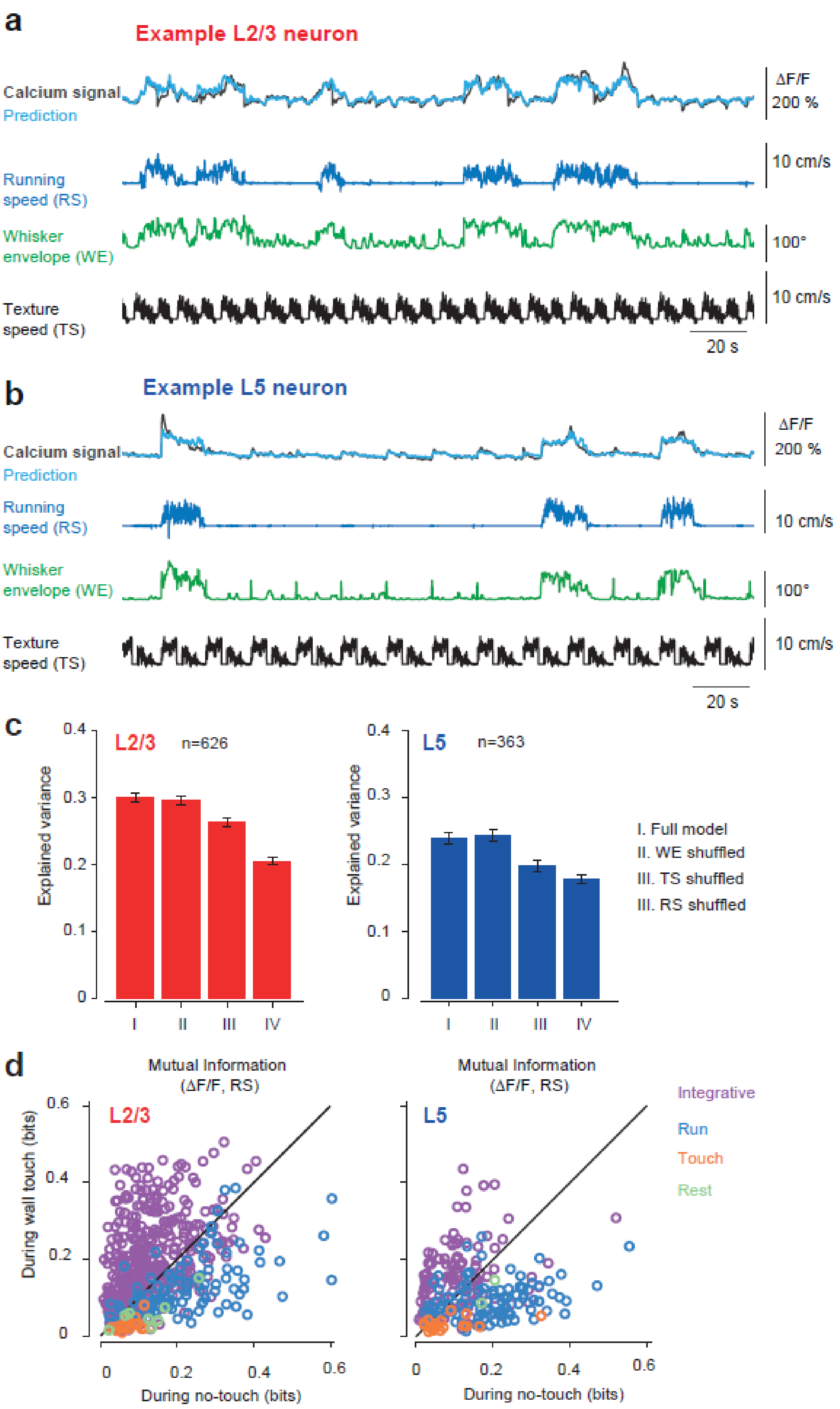

Supplementary Figure 10. Encoding of sensory and motor variables by superficial and deep layer S1 neurons.

(**a**) Prediction of the calcium signal of an example L2/3 neuron based on the motor (run speed in blue, whisking envelope in green) and sensory (texture rotation speed in black) variables using a random forest decoder that was trained and evaluated on separate parts of the data set acquired during a single 'Open-loop' session. Prediction is shown in cyan and actual calcium signal in dark gray. We considered only time periods when texture was in contact with whiskers, so that all three variables were available. (**b**) Same as in **a** but for an example L5 neuron. (**c**) Comparison of prediction quality of four models, I: full model where all variables were available; II: only whisking envelope (WE) shuffled; III: only texture rotation speed (TS) shuffled; and IV: only run speed (RS) shuffled. Red and blue bars show explained variances for L2/3 and L5 neurons, respectively (mean ± s.e.m. are: I: 0.300 ± 0.007, II: 0.296 ± 0.007, III: 0.263 ± 0.007 and IV: 0.206 ± 0.006 for L2/3 and I: 0.239 ± 0.008, II: 0.244 ± 0.008, III: 0.198 ± 0.008 and IV: 0.179 ± 0.007 for L5). We performed paired student's t-test to compare prediction qualities between groups: p(I-II) = 0.69, p(I-III) = 1.06×10^{-4}, p(I-IV) = 2.78×10^{-25} for L2/3; and p(I-II) = 0.69, p(I-III) = 4.58×10^{-4}, p(I-IV) = 4.41×10^{-8} for L5. (a) Mutual information between the calcium signal of each neuron and run speed during 19 Open-loop sessions, where running or resting periods were at least 10% of the time. Mutual information was computed for 'no touch' and 'texture touch' periods separately for L2/3 (left panel, n = 603) and L5 (right panel, n = 233) neurons. Mean mutual information is increased in the presence of touch (0.131± 0.004 vs 0.156 ± 0.004) for L2/3 neurons (p value 2.7×10^{-5}, paired T-test) and decreased (0.148 ± 0.005 vs 0.108 ± 0.003, p = 3.7×10^{-12}, paired T-test) for L5 neurons. Colors of neurons indicate four functional classes of rest (green), run(blue), texture(orange) and integrative(purple) cells as defined in **Fig. 6**. Neurons might be repeated.

REFERENCES

1. Chen, J. L. *et al.* Pathway-specific reorganization of projection neurons in somatosensory cortex during learning. *Nat. Neurosci.* **18,** 1101–1108 (2015).
