# Supplementary material for: Layer-specific integration of locomotion and sensory information in mouse barrel cortex
Source: Nat Commun. 2019 Jun 13;10:2585. doi: 10.1038/s41467-019-10564-8 (PMC6565743; doi:10.1038/s41467-019-10564-8)
Supplement: Supplementary file 3 — Reporting Summary [file 41467_2019_10564_MOESM3_ESM.pdf]

nature research

Corresponding author(s): *Double-blind peer review submissions: write DBPR and your manuscript number here instead of author names.*

Last updated by author(s): *YYYY-MM-DD*

Reporting Summary

Nature Research wishes to improve the reproducibility of the work that we publish. This form provides structure for consistency and transparency in reporting. For further information on Nature Research policies, see Authors & Referees and the Editorial Policy Checklist.

Statistics

For all statistical analyses, confirm that the following items are present in the figure legend, table legend, main text, or Methods section.

n/a	Confirmed	
☐	☒	The exact sample size (n) for each experimental group/condition, given as a discrete number and unit of measurement
☐	☒	A statement on whether measurements were taken from distinct samples or whether the same sample was measured repeatedly
☐	☒	The statistical test(s) used AND whether they are one- or two-sided<br>*Only common tests should be described solely by name; describe more complex techniques in the Methods section.*
☒	☐	A description of all covariates tested
☐	☒	A description of any assumptions or corrections, such as tests of normality and adjustment for multiple comparisons
☐	☒	A full description of the statistical parameters including central tendency (e.g. means) or other basic estimates (e.g. regression coefficient) AND variation (e.g. standard deviation) or associated estimates of uncertainty (e.g. confidence intervals)
☐	☒	For null hypothesis testing, the test statistic (e.g. F, t, r) with confidence intervals, effect sizes, degrees of freedom and P value noted<br>*Give P values as exact values whenever suitable.*
☒	☐	For Bayesian analysis, information on the choice of priors and Markov chain Monte Carlo settings
☒	☐	For hierarchical and complex designs, identification of the appropriate level for tests and full reporting of outcomes
☐	☒	Estimates of effect sizes (e.g. Cohen's d, Pearson's r), indicating how they were calculated

Our web collection on statistics for biologists contains articles on many of the points above.

Software and code

Policy information about availability of computer code

Data collection	Behavior set up was programmed as a custom software written in LabVIEW 2011 (64 bit) using commercial library of functions from Phidgets and Zaber libraries. Two-photon calcium data was collected using HelioScan software.
Data analysis	All data were analyzed in Matlab R2015 (Mathworks) using custom written scripts. The script library for data analysis will be available upon request.

For manuscripts utilizing custom algorithms or software that are central to the research but not yet described in published literature, software must be made available to editors/reviewers. We strongly encourage code deposition in a community repository (e.g. GitHub). See the Nature Research guidelines for submitting code & software for further information.

Data

Policy information about availability of data

All manuscripts must include a data availability statement. This statement should provide the following information, where applicable:

- Accession codes, unique identifiers, or web links for publicly available datasets
- A list of figures that have associated raw data
- A description of any restrictions on data availability

Data will be available upon a reasonable request.

Field-specific reporting

Please select the one below that is the best fit for your research. If you are not sure, read the appropriate sections before making your selection.

☒ Life sciences ☐ Behavioural & social sciences ☐ Ecological, evolutionary & environmental sciences

Life sciences study design

All studies must disclose on these points even when the disclosure is negative.

Sample size	We used 5 mice, 6 field of views (fov) for L2/3 and L5 imaging. Specifically, 'No-touch' condition involved 705 L2/3 and 233 L5 neurons that were imaged over 12 sessions for L2/3 and 8 sessions for L5. Some neurons were imaged multiple times. Total number of unique neurons were 342 and 168 for L2/3 and L5, respectively.<br>In 'Closed loop' condition we imaged 1088 L2/3 neurons in 19 imaging sessions and 728 L5 neurons in 25 sessions. Number of unique neurons were 420 and 275 for L2/3 and L5, respectively.<br>In 'Open-loop' condition we imaged 626 L2/3 and 416 L5 neurons in 11 and 14 sessions respectively. Number of unique neurons were 342 and 236 for L2/3 and L5 respectively.<br>We used all neurons in our analysis, in the case of repeated imaging sessions not to over-represent neurons that were imaged multiple times we used only one instance of imaging sessions for each neuron, unless it is specifically described otherwise.<br>No sample size calculation was performed. The sample sizes are considered adequate for the experiments and consistent with the literature in the field. Statistics are chosen based on the data points and their distribution properties and were clearly stated in each figure.
Data exclusions	Insufficiently motion-corrected trials or time periods within a trial were excluded from the analysis. In rare cases, ROIs with large motion artifacts were also excluded.<br>In addition in Figure 6, 2 sessions were not included where one of the four behavior-stimulus categories were not realized (i.e. there were no periods of texture touch without running). This was clearly stated in the methods.
Replication	Our experimental findings agree with several previous findings:<br>Correlations between running and whisking behavior parameters (response letter figure)<br>Sofroniew, N. J., Cohen, J. D., Lee, A. K. & Svoboda, K. Natural whisker-guided behavior by head-fixed mice in tactile virtual reality. J. Neurosci. 34, 9537–50 (2014).<br>Effect of locomotion on neural responses in barrel cortex (Fig 2):<br>Sofroniew, N. J., Vlasov, Y. A., Hires, S. A., Freeman, J. & Svoboda, K. Neural coding in barrel cortex during whisker-guided locomotion. Elife 4, e12559 (2015)<br>Responses of S1 neurons to rough vs smooth texture (Supplemenatry Fig 6)<br>Chen, J. L. et al. Pathway-specific reorganization of projection neurons in somatosensory cortex during learning. Nat. Neurosci. 18, 1101–1108 (2015).
Randomization	In the case of repeated imaging of a single neuron over several sessisons, we only used data from a randomly selected imaging session of that neuron to avoid over representation of any neuron. In addition statistics of cell category distribution in figure 6 was performed by randomly selecting 11 of 24 sessions 10 times. For each selection percent cells in each category was calculated separately for L2/3 or L5 populations.
Blinding	Investigators were not blinded.

Reporting for specific materials, systems and methods

We require information from authors about some types of materials, experimental systems and methods used in many studies. Here, indicate whether each material, system or method listed is relevant to your study. If you are not sure if a list item applies to your research, read the appropriate section before selecting a response.

Materials & experimental systems

n/a	Involved in the study	
☒	☐	Antibodies
☒	☐	Eukaryotic cell lines
☒	☐	Palaeontology
☐	☒	Animals and other organisms
☒	☐	Human research participants
☒	☐	Clinical data

Methods

n/a	Involved in the study	
☒	☐	ChIP-seq
☒	☐	Flow cytometry
☒	☐	MRI-based neuroimaging

Animals and other organisms

Policy information about studies involving animals; ARRIVE guidelines recommended for reporting animal research

Laboratory animals	Wildtype C57BL6 mice were injected with AAV2.1-EF1α-R-CaMP1.07 for imaging of L2/3 and L5 neurons in barrel cortex (all figures in main text). We used VGAT-Chr2-EYFP mice in Supplementary fig.1 and triple-transgenic mice expressing GCaMP6f in L4 (Nr5a1-Cre;ROSA26-ZtTA;Ai93(TITL-GCaMP6f)) for Supplementary fig.2.
Wild animals	The study did not involve wild animals.
Field-collected samples	The study did not involve field-collected samples.

Ethics oversight	All experiments were conducted in accordance with the ethical principles and guidelines for animal experiments of the Veterinary Office of Switzerland and were approved by the Cantonal Veterinary Office in Zurich.

Note that full information on the approval of the study protocol must also be provided in the manuscript.
