# Supplementary material for: Layer-specific integration of locomotion and sensory information in mouse barrel cortex
Source: Nat Commun. 2019 Jun 13;10:2585. doi: 10.1038/s41467-019-10564-8 (PMC6565743; doi:10.1038/s41467-019-10564-8)
Supplement: Supplementary file 4 — Description of Additional Supplementary Files [file 41467_2019_10564_MOESM4_ESM.pdf]

Description of Additional Supplementary Files

File Name: Supplementary Movie 1
Description: Mouse running/walking in a tactile virtual reality setting. This example video shows a head-restrained mouse running along a textured wall in 'Closed-loop' condition where the rotation speed of the textured wall is coupled to the animal's running speed.

File Name: Supplementary Movie 2
Description: Example whisker tracking video during a 'Closed-loop' trial.
